# Supplementary material for: Rationalisation of Antifungal Properties of α-Helical Pore-Forming Peptide, Mastoparan B
Source: Molecules. 2022 Feb 21;27(4):1438. doi: 10.3390/molecules27041438 (PMC8879275; doi:10.3390/molecules27041438)
Supplement: Supplementary file 1 [file molecules-27-01438-s001.zip › molecules-1497164-Supplementary.pdf]

Figure S1

MIMOTOPES
The Peptide Company

ANALYTICAL DATA

Product Name	2948301
Lot No	P2948301-JQ109385
Column	4.6mm*250mm, Boston Green 0DS-AQ
Solvent A	0.1% trifluoroacetic in 100% acetonitrile
Solvent B	0.1% trifluoroacetic in 100% water

Gradient

	A	B
0.01min	32%	68%
25min	57%	43%
25.1min	100%	0%
30.0min	STOP	

Flow rate	1.0ml/min
Wavelength	220nm
Volume	5ul

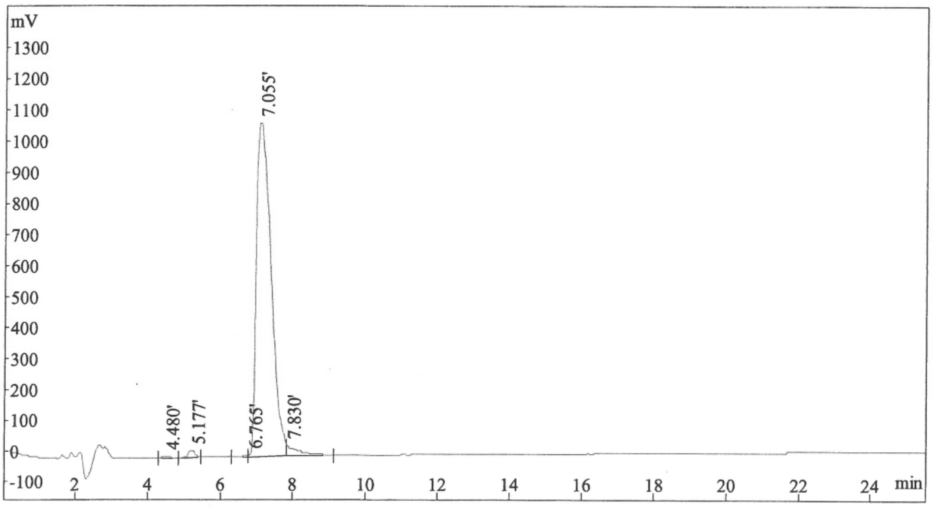

Rank	Time	Conc.	Area	Height
1	4.480	0.1947	58620	5322
2	5.177	0.8218	247420	24788
3	6.765	0.09007	27117	3961
4	7.055	96.46	29040982	1075924
5	7.830	2.433	732545	35608
Total		100	30106684	1145603

Figure S1. RP-HPLC of MB.

Figure S2

MIMOTOPES
The Peptide Company

ANALYTICAL DATA

Product Name	2760501		
Lot No	P2760501-JQ202409		
Column	4.6×250mm, Venusil MP C18-5		
Solvent A	0.1% trifluoroacetic in 100% acetonitrile		
Solvent B	0.1% trifluoroacetic in 100% water		
Gradient		A	B
	0.01min	10%	90%
	25min	67%	33%
	25.1min	100%	0%
	30min		STOP
Flow rate	1.0 mL/min		
Wavelength	214nm		
Volume	5ul		

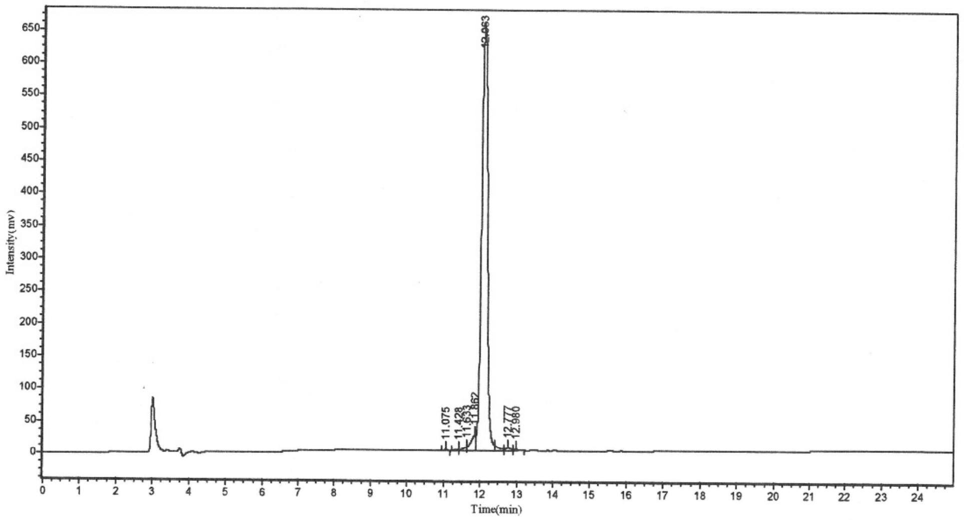

Peak No.	Ret Time	Height	Area	Conc.
1	11.075	940.581	6042.699	0.0757
2	11.428	1039.995	8135.851	0.1019
3	11.633	4490.330	31790.559	0.3982
4	11.862	23532.131	217288.359	2.7219
5	12.063	644091.188	7606070.500	95.2786
6	12.063	8115.689	61754.988	0.7736
7	12.777	3746.849	35188.813	0.4408
8	12.980	1804.731	16704.756	0.2093
Total				100.0000

Figure S2. RP-HPLC of MG.

Figure S3

MIMOTOPES
The Peptide Company

ANALYTICAL DATA

Product Name	3223601		
Lot No	P3223601-JQ817328		
Column	4.6×250mm, Kromasil 100-5C18		
Solvent A	0.1% trifluoroacetic in 100% acetonitrile		
Solvent B	0.1% trifluoroacetic in 100% water		
Gradient		A	B
	0.01min	30%	70%
	25min	97%	3%
	25.1min	100%	0%
	30min	STOP	
Flow rate	1.0 mL/min		
Wavelength	214nm		
Volume	5ul		

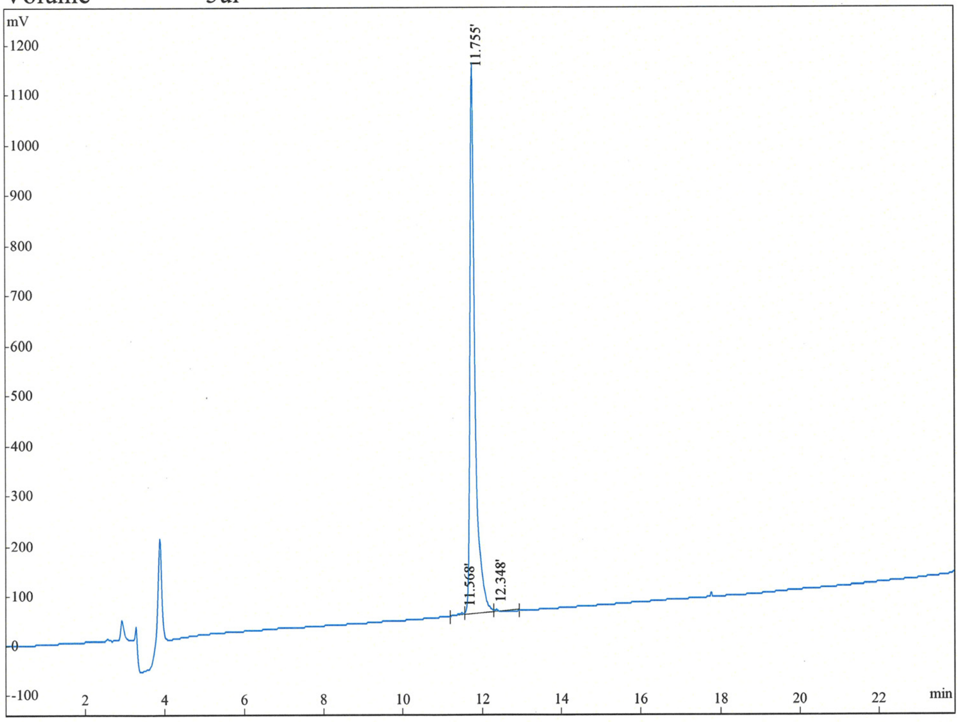

Rank	Time	Conc.	Area	Height
1	11.568	0.5424	52801	4860
2	11.755	98.76	9613368	1112304
3	12.348	0.6992	68062	6637
Total		100	9734231	1123801

Figure S3. RP-HPLC of ML.

Figure S4

MIMOTOPES
The Peptide Company

ANALYTICAL DATA

Product Name	2760503	
Lot No	P2760503-JQ061318	
Column	4.6×250mm,Venusil MP C18-5	
Solvent A	0.1% trifluoroacetic in 100% acetonitrile	
Solvent B	0.1% trifluoroacetic in 100% water	
Gradient	A	B
0.01min	10%	90%
25.00min	67%	33%
25.10min	100%	0%
30.0min	STOP	
Flow rate	1.0ml/min	
Wavelength	214nm	
Volume	5ul	

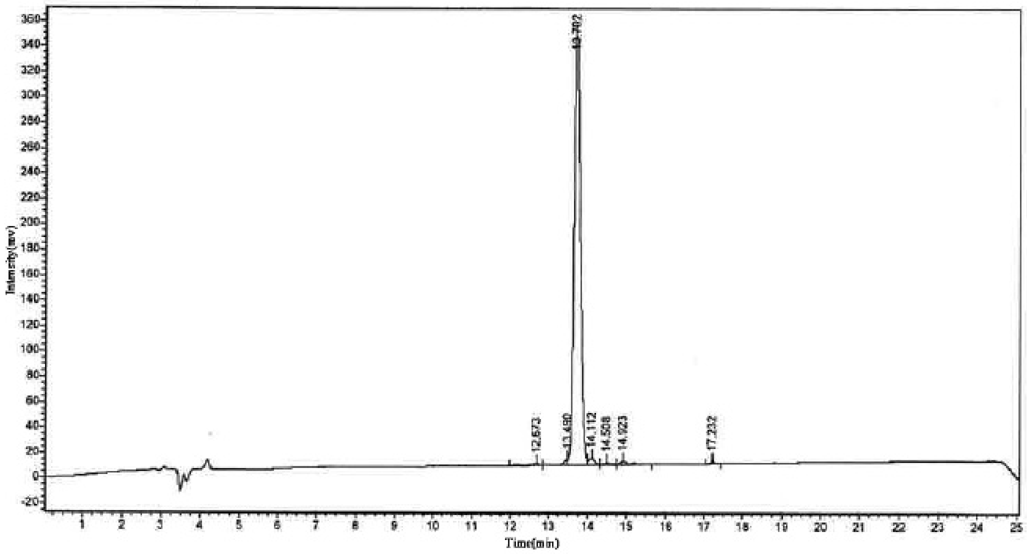

Peak No.	Ret Time	Height	Area	Conc
1	12.673	1057.620	12636.098	0.3175
2	13.490	4214.763	34913.258	0.8773
3	13.702	340328.813	3828297.750	96.2015
4	14.112	4626.406	48164.012	1.2103
5	14.508	1152.984	12833.023	0.3225
6	14.923	2408.923	33715.977	0.8473
7	17.232	1045.157	8896.015	0.2235
Total				100.000

Figure S4. RP-HPLC of CA.

Figure S5

(a) (b)

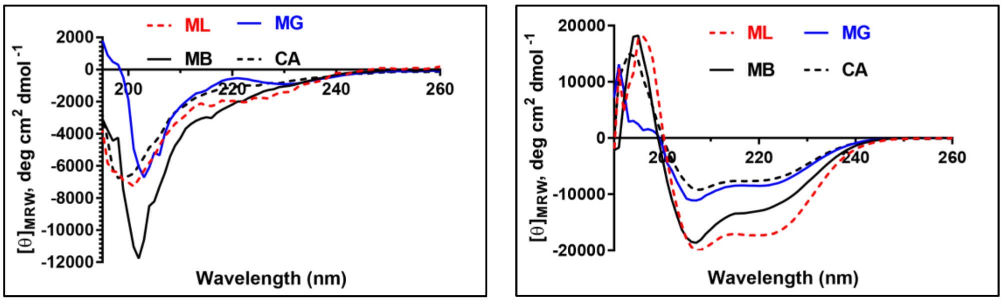

Figure S5. CD spectra of poreforming peptides in (**a**) PBS and in (**b**) 30% TFE. The peptide concentration was 200 μM.
